# 90 Y-ibritumomab tiuxetan: a nearly forgotten opportunity

**DOI:** 10.18632/oncotarget.6531

**Published:** 2015-12-09

**Authors:** Patrizia Mondello, Salvatore Cuzzocrea, Michele Navarra, Michael Mian

**Affiliations:** ^1^ Department of Human Pathology, University of Messina, Messina, Italy; ^2^ Department of Biological and Environmental Sciences, University of Messina, Messina, Italy; ^3^ Lymphoma Service, Memorial Sloan Kettering Cancer Center, New York, NY, USA; ^4^ Department of Hematology, Hospital S. Maurizio, Bolzano/Bozen, Italy; ^5^ Department of Internal Medicine V, Hematology and Oncology, Medical University Innsbruck, Innsbruck, Austria

**Keywords:** radioimmunotherapy, Y-ibritumomab tiuxetan, follicular lymphoma, diffuse large B-cell lymphoma, mantle cell lymphoma

## Abstract

Y-ibritumomab tiuxetan (90Y-IT) combines the benefits of a monoclonal antibody with the efficacy of radiation in the treatment of B-cell non-Hodgkin lymphoma (NHL), a remarkably radiosensitive hematologic malignancy. 90Y-IT activity has been well established in the indolent setting, being approved in front-line treatment of follicular lymphoma (FL) patients as well as salvage therapy. However, no advantage in OS was observed with respect to standard treatment. Promising data are available also for aggressive B-cell lymphoma. In particular, the addition of RIT to short-course first line chemotherapy enables reduction of chemotherapy while maintaining cure rates in elderly, untreated diffuse large B-cell lymphoma (DLBCL) patients. Furthermore, 90Y-IT improves response rate and outcomes of relapsed/refractory DLBCL patients, eligible and ineligible for autologous stem cell transplantation (ASCT). Clinical results have shown a role of 90Y-IT even in mantle cell lymphoma (MCL). RIT might improve responses and treat minimal residual disease when used as consolidation after first-line chemotherapy in MCL. Moreover, 90Y-IT has demonstrated its efficacy in combination with high-dose chemotherapies as conditioning regimen for ASCT, with evidence suggesting the ability to overcome chemotherapy resistance. Herein, we review the available evidence for this approved drug and examine the recently published and ongoing trials for potential novel indication in aggressive B-cell NHL.

## INTRODUCTION

**Figure 1:**
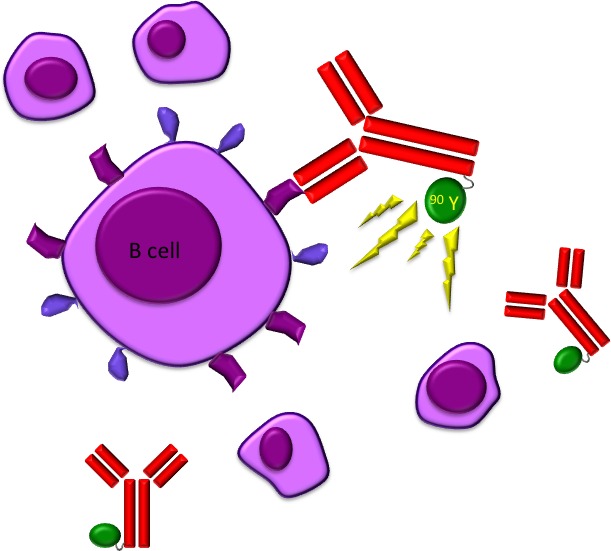
The radio-labeled antibody 90 Y-ibritumomab tiuxetan (Zevalin) binds to cells with CD20 receptors producing a crossfire effect

More than a decade ago, monoclonal antigens targeting B-cell specific antigens to treat B-cell non-Hodgkin lymphomas (NHL) entered clinical routine. [1-3] Nevertheless, in some patients, B-cell lymphomas were a priori resistant to such drugs [4] or developed resistance during therapy. An alternative approach to achieve an additional cytotoxic effect and to overcome some resistance mechanisms was to conjugate monoclonal antibodies to a radioisotope since most lymphomas are highly radiosensitive. In detail, the radiolabeled antibosdy binds to a specific antigen present on cancer cells, bringing the radioactive substance close to the neoplastic cell. This leads not only to death of the bound cancer cell, but also to that of the surrounding cells (“crossfire effect”). (Figure 1) Moreover, radioiummunotherapy (RIT) also induces remodeling of the tumor vasculature favouring neoplasia eradication [4], probably by migration of immune cells towards the malignant lesions [5].

Because CD20 is a transmembrane phosphoprotein expressed in more than 90% of B-cell NHL, in mature B-cells and pre-B cells, but not in plasma cells or stem cells, it represents an attractive target in lymphoma. [6] Currently, there are two approved RIT agents for use in B cell lymphoma: 131 I-tositumomab (Bexxar®; GSK) and 90 Y-ibritumomab tiuxetan (90Y-IT) Zevalin®; Spectrum pharmaceuticals), both targeting CD20. [7, 8] These agents utilize radionuclides that decay by releasing beta particles (high-energy electrons) exerting their lethal effects by causing double-strand DNA breaks in tumor cells.

Bexxar® consists of Iodine-131, a radiation-emitting form of iodine, conjugated to the monoclonal antibody tositumomab, which binds to CD20. lodine-131 emits beta and gamma radiation. Beta radiation is responsible for most of the tumor killing effect while gamma radiation allows gamma camera scans to evaluate the distribution and clearance of radiation from the patient's body. Iodine-131 is eliminated from the body mainly through the urine and by the natural decay of lodine-131.

Instead, Zevalin® consists of indium-111 or yttrium-90 conjugated to ibritumomab which targets CD20. Indium-111 is a gamma emitter used for imaging studies. In November 2011, the FDA considered the biodistribution scan no longer necessary since five prospective trials demonstrated that a true altered biodistribution occurs in approximately only 1% of patients, which is why it is no longer used. [9] Instead, the yttrium-90 radioisotope is a pure beta emitter able to damage and kill the targeted cancer cells as well as nearby cells independently of their CD20 expression. Because of the pure beta emission from yttrium-90 radioisotope, patients do not need any special safety precautions. [10] The situation regarding the special care of patients varies from country to country according to local radio protection laws. For example, Germany and Austria require isolation in a nuclear medicine ward for patients undergoing 90Y-IT treatment. If administered correctly, the mean effective half-life of this drug in blood is 27 hours (range 14-44 h) [11]. Urinary excretion is the primary clearance mechanism, and it accounts for the elimination of almost 10% of unbound 90Y within the first 12 to 24 hours, while the remaining 90% decay. Dosing of 90Y-IT is based on the patient's weight and platelet count. For patients with a platelet count greater than 150,000/μL, the prescribed dose is 0.4 mCi/kg up to a maximum of 32 mCi. For patients with a platelet count between 100,000 to 150,000/μ L, the dose is 0.3 mCi/kg, again up to a maximum of 32 mCi. The most common toxicity for both radioisotopes is myelosuppresion with thrombocytopenia and neutropenia usually begins two to three weeks following the therapeutic dose, remaining low for several weeks and baseline levels being recovered after two to three months. [12] Therefore, the majority of trials investigating RIT activity in B-cell NHL had an inclusion limit of 25% bone marrow infiltration to avoid excessive hematoxicity. Non-haematological toxicities are uncommon and usually minor.

Both agents are currently indicated for the treatment of relapsed or refractory CD20-positive lymphomas, but Bexxar® was withdrawn from the market in October 2013 for commercial reasons. 90Y-IT has an additional treatment indication as consolidation therapy in previously untreated FL patients who achieve a complete response (CR) or a partial response (PR) to first-line chemotherapy.

## FOLLICULAR LYMPHOMA

### Front-line setting

Although FL is characterized by a high overall response rate (ORR) to a single-agent or multidrug immunochemotherapy, relapses are frequent and tend to be less responsive to the next line treatment, which is why advanced stage FL is still considered as incurable with the current standard of care [13]. Moreover, after the first relapse, the time to next recurrence is shorter leading to a median overall survival of 4-5 years. For these reasons, achieving CR or even elimination of minimal residual disease (MRD), is more advisable in first line than with the following treatment lines. Several authors investigated the administration of 90Y-IT either as consolidation treatment after immunochemotherapy or as monotherapy in first line Table 1.

**Table 1:** 90Y-ibritumomab tiuxetan treatment in follicular lymphoma

Trial design	Disease	No of patients	Response rate	Response duration
**After induction therapy [15]**	Chemonaïve FL	208	CR 87%	mPFS 49 months (all responder) 92 months (in CR) 30 months (in PR)
**After 3cycles of R-CHOP/R-CVP [16]**	Chemonaïve FL	41	CR 72%	5-year PFS 64% 5-year OS 96%
**After 3 cycles of R-CHOP [17]**	Chemonaïve FL	60	CR 96%	2-year PFS 78% 2-year OS 100%
**After 4 cycles of R-FND [18]**	Chemonaïve FL	47	CR 91%	5-year PFS 74% 5-year OS 93%
**After 4 cycles of FM [19]**	Chemonaïve FL and MZL	22	ORR 100% CR 79% PR 21%	mPFS 47.2 months mOS not reached
**After 4 cycles of R-FM [20]**	Chemonaïve FL	55	ORR 96% CR 89%	3-year PFS 81% 3-year OS 100%
**Monotherapy [23]**	Chemonaïve FL	59	ORR 87% CR 56% PR 31%	mPFS 25.9 months
**Monotherapy [26]**	Chemonaïve FL	74	ORR 96% CR 69%	3-year PFS 58% 3-year OS 95%
**Monotherapy [27]**	R/R FL	54	ORR 74% CR 50%	mTTP 6.8 months
**Monotherapy [28]**	R/R FL	143	ORR 80% CR 20%	mTTP 12.6 months
**Monotherapy [29]**	R/R FL	211	ORR eR 86% lR 72% CR eR 51% lR 28%	mTTP eR 15.4 months lR 9.2 months

**Abbreviations:** FL follicular lymphoma; CR complete response; PR partial response; mPFS median progression-free survival; OS overall survival; ORR overall response rate; mTTP median time to progression; R/R relapsed/refractory; R-CHOP rituximab, cyclophosphamide, doxorubicin, vincristine and prednisone; R-CVP rituximab, cyclophosphamide, vincristine and prednisone; R-FND rituximab, fludarabine, mitoxantrone, dexamethasone; FM fludarabine, mitoxantrone; eR early relapse; lR late relapse.

#### RIT as consolidation after induction therapy

In FL, as in other hematologic neoplasias, MRD is becoming always more important since it significantly improves the outcome [14]. Therefore, RIT as consolidation after induction immunochemotherapy could induce a more profound response, postponing an eventual relapse or even lead to cure of the disease. Indeed, the FIT randomized phase III trial [15] proved a benefit for 90Y-IT as consolidation in previously untreated FL patients. After completing induction therapy of investigators's choice, patients were randomized to receive either standard dose of 90Y-ibritumomab tiuxetan (*n* = 208) or no further treatment (*n* = 206). The regimens used were cyclophosphamide, vincristine and prednisone (CVP/COP) (*n* = 106), anthracyclin-based regimens (*n* = 183), fludarabine-based therapies (*n* = 22), chlorambucil (*n* = 39) and rituximab in association with chemotherapy (*n* = 59). After (immuno-)chemotherapy, the CR rate was similar in both arms (53% in the observation arm and 52% in the 90Y-IT arm). After RIT almost three-quarters of patients in PR converted to CR, leading to CR rate of 87%. This response improvement led to a significant PFS prolongation of more than 2 years in the RIT-consolidation arm as compared to the control arm. In detail, PFS was 49 *vs* 15 months in all responders, 30 *vs* 6 months for partial responders and 92 *vs* 32 months in complete responders, suggesting that 90Y-IT improves PFS regardless of response to therapy. Moreover, 90Y-IT was able to prolong median time to next treatment (TTNT) for more than 5-years in comparison to the control group. However, there was no statistical significant difference in OS, probably due to the usually indolent clinical course of FL. Despite these promising results, their value for current clinical routine is limited since almost all patients nowadays receive rituximab in first line, while in the FIT trial approximately only 14% underwent immunochemotherapy. This because at the time the study was designed rituximab was not yet a standard agent for front-line therapy in some countries involved int the trial. Due to the small number of rituximab-treated patients, the subgroup analysis including only those patients was insufficiently powered to detect a difference in final CR rates (93% for the 90Y-IT arm *vs* 71% for the observation arm) or in rates of conversion from PR to CR (71% *vs* 42%, respectively) despite promising results.

The use of RIT as first line consolidation might also allow for the reduction of the number of immunochemotherapy cycles. Five studies investigated 90Y-IT consolidation after abbreviated courses of rituximab-based induction regimens. Hainsworth et al. [16] conducted a phase II trial including 41 patients with previously untreated FL who received rituximab for 4 consecutive weeks, followed by 3 cycles of rituximab combined with either CHOP (88%) or CVP (12%) prior to 90Y-IT consolidation. 90Y-IT was able to increase the CR rate from 30% to 72%. After a median follow-up of 67 months, the estimated 5-year PFS and OS rates were 64% and 96% (*p* = 0.3), respectively. Another phase II trial [17] included 60 patients with stage II-IV symptomatic or bulky FL who received three cycles of R-CHOP followed by RIT and four additional weekly rituximab administrations. The percentage of CR assessed with positron emission tomography (PET), improved from 46% after induction therapy to 89% following 90Y-IT consolidation. The remaining three trials [18-20] investigated the efficacy of fludarabine-based immunochemotherapy regimens plus 4 or more cycles of rituximab followed by RIT consolidation, with or without adjuvant rituximab maintenance, in untreated and intermediate/high risk FL patients. The conversion rates from PR to CR after RIT varied from 13% to 95%, conferming that the addition of RIT consolidation to immunochemotherapy was able to improve the quality of responses as well as long-term outcomes, even for patients previously treated with rituximab. Therefore, 90Y-IT is an interesting treatment option for a subgroup of FL pateints, for example those who are not eligible for rituximab maintenance. [21, 22]

#### RIT monotherapy in first-line treatment

90Y-IT has proved to be an efficient and feasible treatment option as front-line therapy of FL. Scholz et al. [23] evaluated the efficacy and safety of RIT in 59 chemonaive, fit FL patients. The ORR at 6 months after RIT was 87%, with 41% of the patients achieving CR, 15% CR unconfirmed (CRu), and 31% PR. Median PFS was 25.9 months. Compared with the standard first line immunochemotherapies, the CR rates achieved with 90Y-IT are similar [24], while median PFS was below the reported values [24, 25]. However, patients in CR after RIT alone in first line achieved long-lasting remission with a PFS not reached after a follow-up of nearly 31 months. 90Y-IT might delay or even avoid the need for chemotherapy in first line for a considerable number of patients, leaving aggressive regimens an option for relapsed FL. Furthermore, 90Y-IT as first line treatment has achieved a superior ORR with respect to four courses of rituximab monotherapy in the same setting. [23] In all trials, RIT was safe with the most common adverse event being transient myelosuppresion. Non-hematologic toxicities were unfrequent and never exceeded grade 2.

The favourable toxicity profile and the efficacy of 90Y-IT were also confirmed in a more recent phase II trial [26] evaluating a fractionated 90Y-IT administration (11.1MBq/kg given 8-12 weeks apart) in 74 untreated, fit FL patients. The ORR was 96% with 69% CR/CRu. After a median follow-up of 3.1 years, PFS was 58%, treatment-free survival 66%, and OS 95%. Noteworthy, in this study the patient cohort had a more unfavourable baseline characteristics profile with respect to the previous one [23] with almost twice as many high-risk FLIPI patients (44%), but displayed a superior median PFS (40.2 v 26 months). In addition, 3-year PFS of 85% (median not reached) for patients with CR was markedly improved compared with the 1- and 2-year PFS rates of 77% and 54% reported by Scholz [23]. No significant difference in PFS was observed for patients with or without bulky disease, suggesting that patients with large initial tumor bulks may benefit from fractionation of the therapy. Tumor regression after the initial 90Y-IT infusion may allow improved delivery to sites of bulk with the second fraction. [26] The median PFS of 40.2 months is in line with the one after non-anthracycline-based regimen (eg. R-CVP), but slightly inferior to R-CHOP and R-Bendamustine [24, 25]. However, fractionated RIT led to high response rates as initial treatment in a high-risk population with one third of patients achieving CR and durable remissions. Consequently, RIT might be an interesting strategy for a selected subpopulation, not eligible for chemotherapy.

Therefore, RIT has an important part in the front line setting, either as single agent or as consolidation following standard chemoimmunotherapy. RIT as single agent showed to be superior with respect to the immunotherapy, which is why it should be considered in patients who are not candidates for standard (immuno-)chemotherapy. 90Y-IT consolidation is superior to observation following standard immunochemotherapy, increasing the conversion of PR to CR, prolonging relapse free survival and improving OS.

### Relapsed/refractory FL

Several studies have demonstrated that 90Y-IT is safe in patients who underwent more treatment lines with relapsed or refractory low-grade lymphoma (Table 1) [27-29]. Witzig et al. [27] administered 90Y-IT at a dose of 0.4 mCi/kg to 54 FL patients, refractory to rituximab, obtaining an ORR of 74% with 50% of CR, and median time to progression of nearly 7 months. Based on these results, Gordon et al. [28] randomized 143 rituximab-naive patients with relapsed or refractory FL or transformed B-cell NHL to receive either 90Y-IT or rituximab alone. 90Y-IT proved to be more efficient in inducing a response than rituximab in this patient setting, leading to an ORR and a CR rate of 80 *vs* 56% (*p* = 0.002) and 30 *vs* 16% (*p* = 0.04), respectively. Although this study was not powered to detect differences in time dependent variables, there was a trend towards a longer median time to progression (TTP; 15 *vs*. 10.2 months; *p* = 0.07), duration of response (16.7 *vs*. 11.2 months; *p* = 0.44) and time to next therapy (21.1 *vs*. 13.8 months; *P* = 0.27). Interestingly, in patients achieving a CR after 90Y-IT, the median TTP was longer, though it was not significant when compared to the control group (24.7 months v.s. 13.2 months; *p* = 0.41), suggesting a more profound disease eradication. As observed in other anticancer treatments, the administration of 90Y-IT in an earlier treatment line led to a better outcome [29]. Pooled data from four clinical trials including patients with relapsed FL demonstrated a significantly higher ORR (86% *vs* 72%; *p* = 0.051), CR rate (51% *vs* 28%; *p* = 0.004) and longer TTP (12.6 *vs* 7.9 months, *p* = 0.038) when 90Y-IT was administered in first relapses compared to a higher treatment line. Therefore, RIT represents a valid approach for relapsed or refractory FL patients as well, especially in elderly or unfit patients who can not undergo transplant or aggressive regimens.

## DIFFUSE LARGE B-CELL LYMPHOMA

90Y-IT has been evaluated as consolidation after first line therapy and in relapsed or refractory patients as part of transplantation setting (Table 2).

**Table 2:** 90Y-ibritumomab tiuxetan treatment in diffuse large B cell lymphoma

Trial design	Disease	No of patients	Response rate	Response duration
**After 6 cycles of CHOP [30]**	Chemonaïve DLBCL	20	ORR 100% CR 95% PR 5%	2-year PFS 75% 2-year OS 95%
**After 6 cycles of R-CHOP [31]**	Chemonaïve, elderly DLBCL	63	ORR 88% CR/CRu 86% PR 2%	3.5-year PFS 75% 3.5-year OS 84%
**After 4 cycles of R-CHOP [32]**	Chemonaïve, elderly DLBCL	55	ORR 80% CR 73% PR 7%	2-year PFS 85% 2-year OS 86%
**After either 4 or 6 cycles of R-CHOP [34]**	Chemonaïve, early stage DLBCL	53	ORR 98% CR/CRu 79% PR 19%	5-year PFS 78% 5-year OS 94%
**Monotherapy [37]**	R/R DLBCL	104	ORR 44%	mPFS 1.6 - 5.9 months mOS 4.6 – 22.4 months
**Monotherapy followed by 4 weekly doses of rituximab [39]**	R/R DLBCL	25	ORR 31% CR 21% PR 13%	mEFS 2.5 months mOS 8.1 months 5-year OS 53% (all patients) 81% (long-term responders)
**Z-BEAM [40]**	R/R DLBCL	43	ORR 98% CR 98%	2-year PFS 59% 2-year OS 91%
**Z-BEAM [41]**	Transformed lymphoma	63	ORR 100%	2-year PFS 68% 2-year OS 90%
**After 3 cycles of R-CHOP and in association with ASCT [42]**	DLBCL patients in PR or CRu after first line treatmen	37	ORR 100%	mPFS 5.1 years mDFS 4.3 years mOS 7.8 years

**Abbreviations:** DLBCL diffuse large B cell lymphoma; ORR overall response rate; CR complete response; PR partial response; mPFS median progression-free survival; mOS median overall survival; R-CHOP rituximab, cyclophosphamide, doxorubicin, vincristine and prednisone; R/R relapsed/refractory; mEFS median event free survival; BEAM carmustine, etoposide, cytarabine, and melphalan; ASCT autologous stem cell transplantation; mDFS median disease free survival.

### RIT as consolidation after induction therapy

RIT has been studied as consolidation after CHOP-based therapy in untreated elderly DLBCL patients [30-32]. Zinzani et al. [30] administered a 90Y-IT consolidation to 20 patients after 6 cycles of CHOP. Four out of 5 patients in PR after CHOP achieved a CR, leading to an overall CR rate of 95% and PR of 5%. The 2-year PFS was 75% with a 2-year OS of 95%. However, CHOP is not the standard first line therapy anymore [33] and the addition of 90Y-IT seems to have similar results with respect to R-CHOP. Since both drugs target CD20, the prior use of rituximab in induction treatment could hamper the efficacy of RIT after R-CHOP. Nevertheless, the patient group used was small and consequently it was not possible to draw a conclusion from this study.

In a phase II study 63 high-risk elderly, untreated DLBCL patients, who were ineligible for stem cell transplantation, underewent 6 cycles of R-CHOP and then those with responding or stable disease received 90Y-IT consolidation 6-9 weeks later. After completion of the induction treatment, 50 patients were eligible for 90Y-IT and 44 were ultimately treated. 90Y-IT was well tolerated and 86% of patients achieved a CR/CRu, 2% experienced a PR, while 12% did not respond. 90Y-IT improved response from PR to CR or CRu to CR in 16% of patients. At 42 months, the OS for 90Y-IT treated patients was 83.5% and the PFS was 74.5%. [31]

Based on these promising data, a phase II trial evaluated the possibility to administer a short-course R-CHOP chemotherapy followed by 90Y-IT in order to increase the global treatment efficacy along with a decreased exposure to cytotoxic drugs [32]. 55 high-risk elderly DLBCL patients received four courses of R-CHOP and 48 underwent RIT. 90Y-IT improved the remission status of 8/16 patients in PR after R-CHOP regimen leading to a ORR of 80% (CR 73%, PR 7%). The 2-year PFS was 85% with 2-year OS of 86%. These promising results provided the rationale for a randomized, phase III trial, which aims at evaluating the value of RIT after R-CHOP or R-CHOP-like therapy in a large cohort of elderly untreated, DLBCL patients (NCT01510184).

Recently, Witzig et al. [34] have evaluated the role of 90Y-IT in early stage DLBCL. Fifty-three patients underwent either four (21/53, 40%) or six (30/53, 57%) cycles of R-CHOP followed by RIT. After induction immunochemotherapy, the ORR was 98% (CR/CRu 79%, PR 19%). Forty-eight patients proceeded to RIT. Four of the five cases with CRu undergoing to RIT converted to CR. Of the 10 patients in PR, three achieved a CR, one a CRu while three did not change their status remission and one progressed during RIT. At 5-year follow-up, PFS was 78% and OS was 94%. Among 52 responders 84% remained in remission after 5-years. 90Y-IT demonstrated to improve the ORR and reduce relapses in this setting. These results are comparable with the two Southwestern Oncology Group (SWOG) studies for early stage DLBCL. Treatment consists in the SWOG0014 [35] in 3 cycles of R-CHOP and involved field radiation therapy (IFRT) while in the SWOG0313 [36] in 3 cycles of CHOP followed by IFRT and then 90Y-IT. Unlike the SWOG studies, the ECOG3402 [34] demonstrated that adding RIT may enable reduction in chemotherapy for patients at least in PR at ther interim restaging while maintaining cure rates. However, in the ECOG3402 trial, interim response was evaluated by computered tomography (CT), while in clinical practice PET is preferred. For this reason, the SWOG S1001 phase II trial is currently evaluating a PET guided treatment approach in patients with limited-stage DLBCL. After 3 cycles of R-CHOP chemotherapy, all patients will be restaged by PET/CT. Patients with a CR will receive one additional cycle of R-CHOP, for a total of 4 cycles. Those with a PR will receive standard dose involved-field radiotherapy followed by a single infusion of 90Y-IT. (NCT01359592)

RIT might have a role as consolidation after induction therapy in elderly or unfit DLBCL patients, allowing for the reduction of the amount of chemotherapy and to maintain treatment efficacy. Results from the current clinical trial will help to clarify if the combination therapy is better than 6 full cycles of R-CHOP therapy and whether it would be advisable in selective setting of patients.

### Relapsed/refractory DLBCL

RIT has demonstrated to be also an efficient and safe treatment option for relapsed or refractory DLBCL patients, achieving promising response rates and durable disease remissions. [27, 37-39] Morschhauser et al. [37] evaluated the efficacy of 90Y-IT in 104 DLBCL patients who were either refractory or relapsed after first line therapy associated or not with rituximab. The ORR was 44% and, as expected, it was higher in patients without a prior rituximab administration compared to those who underwent immunochemotherapy as primary treatment (53% *versus* 19%, respectively). The median PFS in three groups ranges from 1.6 to 5.9 months. Arnason et al. [39] confirmed the potential role of RIT in this poor risk subset. 90Y-IT was administered in 25 relapsed/refractory DLBCL patients, not candidates for transplant, followed by 4 weekly doses of rituximab. All non-progressing patients received 4 weekly doses of maintenance rituximab every 6 months for 4 cycles. The ORR was 31% (CR 21%, PR 13%). Median event free survival (mEFS) was 2.5 months. Median OS was 8.1 months. In addition, a study evaluating data from 4 clinical trials using 90Y-IT in recurring NHL demonstrated that RIT can produce durable responses and prolonged overall survival in a substantial number of patients in whom previous therapies have failed. The 5-year OS was 53% for all patients and 81% for long-term responders. Therefore, RIT might be considered as a valid therapeutic option for relapsed/refractory patients that refuse or can not tolerate chemotherapy. Indeed, RIT has response rate and survival similar to other chemotherapy regimens currently available. Furthermore, RIT is generally feasible and requires less time to complete the treatment.

90Y-IT in association with high-dose chemotherapy followed by ASCT proved to be efficient and safe in heavily pretreated patients as well. [40-42] A phase II trial randomized 43 relapsed/refractory DLBCL patients to receive high-dose chemotherapy consisting in carmustine, etoposide, cytarabine, and melphalan (BEAM) alone (*n* = 21) or combined to 90Y-IT (Z-BEAM, *n* = 22). Although the PFS difference between the two treatment arms was not statistically significant (59% and 37% after Z-BEAM and BEAM alone, respectively; *p* = 0.2), two-year OS was 91% and 62% after Z-BEAM and BEAM, respectively (*p* = 0.05). [40] Mei et al. reported encouraging results using the Z-BEAM conditioning regimen in 63 patients with transformed low grade lymphoma, which usually has a very poor prognosis [41]. Two-year PFS was 68%, and OS was 90%. A recent meta-analysis has shown that Z-BEAM improves PFS and OS at 2-years (*p* < 0.05 and *p* < 0.01) with respect to BEAM. In addition, 90Y-IT could overcome a poor pretransplant response rate without further toxicities. [43] Based on these promising results, a randomized phase II trial which compares R-BEAM with or without 90Y-IT in DLBCL patients eligible for transplantation is currently ongoing. (NCT00591630). This confirmatory trial will determine the real advantage of adding 90Y-IT to high-dose chemotherapy and may drive a change in the standard conditioning regimen to use in clinical practise.

The role of 90Y-IT as consolidation after early salvage transplant treatment was retrospectively evaluated. Thirty-seven patients with intermediate-high risk DLBCL not in CR assessed by PET after three cycles of R-CHOP switched to high dose chemotherapy followed by ASCT. Twenty patients underwent additional consolidation with ^90^Y-IT. At the end of the treatment CR rate converted from 45% to 100%. PFS (5.1 years *versus* 2.7 years, *p* = 0.007) and DFS (4.3 years *versus* 2.0 years, *p* = 0.001) were significantly longer in the ^90^Y-IT group. However, no difference in overall survival was observed, [42] but these are retrospective data. Currently there are no ongoing studies to confirm this finding and, therefore, this approach remains experimental.

## MANTLE CELL LYMPHOMA

90Y-IT has been investigated as consolidation after first line therapy, as part of transplantation setting and in relapsed or refractory patients (Table 3).

**Table 3:** 90Y-ibritumomab tiuxetan treatment in mantle cell lymphoma

Trial design	Disease	No of patients	Response rate	Response duration
**After 4 cycles of R-CHOP [48]**	Chemonaïve MCL	56	ORR 88% CR 67%	mTTF 34.3 months mPFS 31 months 2-years OS 90%
**Z-BEAM [55]**	R/R NHL	44	ORR 73%	3-year PFS 43% 3-year OS 60%
**Z-BEAM [53]**	R/R NHL	41	ORR 66%	2-year PFS 68% 2-year OS 85%
**Z-BEAM or BEAC [56]**	Chemonaïve MCL	162	ORR 96% CR/CRu 91% PR 4%	4-year PFS 71%
**Z-BEAM [57]**	R/R MCL	46	ORR 100% CR 36% PR 64%	4-year PFS 41% 5-year OS 71%
**After ASCT (unpublished data)**	Chemonaïve MCL	57	ORR 100% CR 100%	5-year PFS 79% 5-year OS 96%
**Monotherapy [58]**	R/R MCL	34	ORR 67% CR 15%	OS 21 months mEFS 6 months
**Monotherapy [62]**	R/R MCL	6	ORR 50% PR 33% SD 16%	mPFS 3.9 months
**Combined with bortezomib [66]**	R/R MCL	12	ORR 50% CR 42%	mPFS 6.4 months (all patients) 23 months (patients in CR) 4.4 months (patients in PR/SD)

**Abbreviations:** R/R relapsed/refractory; MCL mantle cell lymphoma; NHL non Hodgkin lymphoma; ORR overall response rate; CR complete response; PR partial response; mPFS median progression-free survival; OS overall survival; mTTF median time to treatment failure; mEFS median event-free survival; mDFS median disease free survival; R-CHOP rituximab, cyclophosphamide, doxorubicin, vincristine and prednisone; BEAM carmustine, etoposide, cytarabine, and melphalan; ASCT autologous stem cell transplantation.

### Front-line setting

#### RIT as consolidation after induction therapy

R-CHOP as initial therapy for untreated MCL yields a high response rate, but remissions are not durable [44, 45] Because MCL is predominantly a disease of patients older than 60 years, a valid approach to improve R-CHOP outcomes is to add a consolidation strategy. Because of the activity of RIT in MCL [46, 47], its favorable toxicity profile, and the need for a consolidation treatment applicable to all patients, including older and less-fit patients, the ECOG E1499 trial [48] investigated the efficacy of 4 cycles of R-CHOP chemotherapy followed by 90Y-IT consolidation in 56 newly diagnosed stage II-IV MCL. The ORR was 88% at completion of all therapy, with a CR rate of only 13% after R-CHOP but 67% after 90Y-IT. Time to treatment failure (TTF) was 34.2 months. This median TTF is better than that previously reported for six cycles of R-CHOP. [25, 44]. The 2-years OS rate was 90%, with an mPFS of 31 months. Alternative strategies to improve R-CHOP results include maintenance. Rituximab maintenance after R-CHOP regimen has demonstrated to prolong response duration, achieving a PFS from 37 to 56 months [49, 50]. An interesting question would be the comparison of RIT consolidation followed by rituximab maintenance, with rituximab maintenance alone.

Currently, in untreated MCL patients who cannot tolerate more-intensive regimens or clinical trials, R-CHOP for four cycles followed by 90Y-IT could be a reasonable approach as initial therapy.

#### RIT-based stem cell transplant regimens for MCL

Another approach to improve outcomes after R-CHOP is to add consolidation therapy with high-dose chemotherapy followed by ASCT. [51] Incorporating RIT into a transplant regimen might further enhance eradication of residual disease after induction therapy and ultimately prolong PFS and OS. [52-57]

A phase I dose-finding trial [55] of escalated dose RIT followed by high dose chemotherapy with BEAM and autologous stem cell reinfusion established the maximum-tolerated radiation-absorved dose (RAD) to critical organs as 15Gy. In this cohort, seven out of 44 patients (16%) were affected by MCL. Among the 29 patients, (66%) with active disease at study entry, 11 achieved CR, and 6 achieved PR after protocol treatment. The estimated 3-year PFS and OS rates were 43% and 60%, respectively. There was no difference in PFS and OS between the different histologic subsets.

Based on these encouraging data, a phase II trial [53] associating 90Y-IT with BEAM before ASCT was conducted in patients with refractory, relapsed or poor-risk NHL. The 2-year PFS and OS in the 13 patients included with MCL were 68% and 85%, respectively. In order to improve outcome for patients not in CR before ASCT the Nordic Lymphoma Group added 90Y-IT to the high-dose induction chemotherapy. In the multicenter phase 2 trial MCL-3 study [56] patients in PR or CRu after first line treatment were randomized to receive 90 Y-IT in combination with BEAM or BEAC (C = cyclophosphamide) before ASCT. Although Z-BEAM followed by ASCT was feasible and without additional toxicity, it was not associated with improved PFS, probably because intensification with 90Y-IT may be too late to improve the outcome in patients not in CR before transplant. Similarly, a recent retrospective study [57] has demonstrated that Z-BEAM followed by ASCT in relapsed or refractory MCL patients is not associated with significant survival improvement compared to the standard rituximab and BEAM (R-BEAM). The 5-year OS was 55% and 71% (*p* = 0.2) in the R-BEAM *vs* Z-BEAM groups, and the 4-year PFS was 32% and 41% (*p* = 0.3), respectively. Furthermore, we retrospectively asessed the role of 90Y-IT as consolidation after ASCT in first line (data not yet published). Treatment intensification was well tolerated and led to a significantly longer response duration in comparison to the standard treatment (PFS was not reached *versus* 7 years, *p* = 0.001; OS was not reached *versus* 8.1 years, *p* = 0.008). The 5-year PFS was 79% in the 90Y-IT group compared to 55% in the other one. The 5-year OS was superior in the 90Y-IT group with respect to the other one as well (96% *versus* 81%). In contrast to the historical cohort, the addition of 90Y-IT seemed to overcome important risk factors such as MIPI and bone marrow infiltration. Consolidation with ^90^Y-IT after induction and ASCT seems not only able to significantly reduce the number of disease recurrences but also to delay relapse in newly diagnosed MCL patients with intermediate/high MIPI score.

The role of RIT as consolidation of high-dose chemotherapy is unclear. Because of the contrasting results there is no reason to change the clinical practice. Currently, several trials evaluating RIT followed by autologous or allogenic stem cell transpant are ongoing (NCT00505232, NCT00695409, NCT00607854, NCT01434472) and will help to clarify this issue.

### Relapsed/refractory MCL

Despite the intensification of first line treatment, relapses remain frequent [44, 45]. Wang et al. [58] reported a phase II trial using 90Y-IT as single agent in 34 refractory or relapsed MCL patients. All patients were heavily pretreated with a median of three prior chemotherapy regimens (range 1-6). ORR was 67% with 15% of CR rate. At median follow-up of 22 months, median OS was 21 months and mEFS was 6 months. This single-agent activity compared favorably with that of other single agents. [59-61] In particular, the remission rates and progression free survival duration obtained in patients who had previously received rituximab are similar to previous experiences with bortezomib and temsirolimus [59, 61]. In addition, 90Y-IT was more convenient due to the short time required to finish the treatment (1-2 weeks) rather than protracted duration. Morover, 90Y-IT had fewer adverse effects than these agents. A similar study using 90Y-IT in relapsed/refractory MCL patients obtained comparable single-agent activity. [62] ORR was 50% with 33% of PR rate and 16% of SD. PFS was 3.9 months. However, these results are not really satisfactory. Based on preclinical data demonstrating synergy between proteasome inhibitor and radiation [63-65], a more recent phase I study evaluated the safety of 90 Y-IT combined with bortezomib in patients with relapsed or refractory MCL. The ORR was 50% with 42% of CR rate. [66] Given these promising results, a phase II trial is currently evaluating the efficacy of this novel combination in relapsed or refractory MCL. (NCT01497275)

## CONCLUSIONS

90Y-IT is an effective and safe drug, which combines the benefits of a monoclonal antibody with the efficacy of radiation in the treatment of B-cell NHL, a remarkably radiosensitive hematologic malignancy. 90Y-IT activity has been well established in the indolent setting as supported by the FDA approval for treatment of chemonaive and relapsed/refractory FL patients. Nevertheless, no advantage in OS was reported with respect to the standard. The efficacy of RIT has been observed in aggressive NHL as well. However, future randomized, clinical trials are warranted to clarify the role of RIT in this setting and support its approval as alternative therapeutic approach.
